# Fluorescence grid analysis for the evaluation of piecemeal surgery in sinonasal inverted papilloma: a proof-of-concept study

**DOI:** 10.1007/s00259-021-05567-x

**Published:** 2021-11-05

**Authors:** J Vonk, FJ Voskuil, JG de Wit, WT Heeman, WB Nagengast, GM van Dam, RA Feijen, AGW Korsten-Meijer, B van der Vegt, MJH Witjes

**Affiliations:** 1 Department of Oral & Maxillofacial Surgery, University of Groningen, University Medical Center Groningen, PO Box 30.001, 9700 RB Groningen, the Netherlands; 2 Department of Pathology & Medical Biology, University of Groningen, University Medical Center Groningen, Groningen, the Netherlands; 3 Medical Imaging Center, University of Groningen, University Medical Center Groningen, Groningen, the Netherlands; 4 Faculty Campus Fryslân, University of Groningen, Leeuwarden, the Netherlands; 5 LIMIS Development B.V, Leeuwarden, the Netherlands; 6 Department of Gastroenterology and Hepatology, University of Groningen, University Medical Center Groningen, Groningen, the Netherlands; 7 AxelaRx/TRACER B.V, Groningen, the Netherlands; 8 Department of Otorhinolaryngology/Head and Neck Surgery, University Medical Center Groningen, University of Groningen, Groningen, the Netherlands

**Keywords:** Paranasal sinus neoplasms, Papilloma, Inverted, Optical imaging, Molecular imaging, Vascular endothelial growth factor A

## Abstract

**Purpose:**

Local recurrence occurs in ~ 19% of sinonasal inverted papilloma (SNIP) surgeries and is strongly associated with incomplete resection. During surgery, it is technically challenging to visualize and resect all SNIP tissue in this anatomically complex area. Proteins that are overexpressed in SNIP, such as vascular endothelial growth factor (VEGF), may serve as a target for fluorescence molecular imaging to guide surgical removal of SNIP. A proof-of-concept study was performed to investigate if the VEGF-targeted near-infrared fluorescent tracer bevacizumab-800CW specifically localizes in SNIP and whether it could be used as a clinical tool to guide SNIP surgery.

**Methods:**

In five patients diagnosed with SNIP, 10 mg of bevacizumab-800CW was intravenously administered 3 days prior to surgery. Fluorescence molecular imaging was performed in vivo during surgery and ex vivo during the processing of the surgical specimen. Fluorescence signals were correlated with final histopathology and VEGF-A immunohistochemistry. We introduced a fluorescence grid analysis to assess the fluorescence signal in individual tissue fragments, due to the nature of the surgical procedure (i.e., piecemeal resection) allowing the detection of small SNIP residues and location of the tracer ex vivo.

**Results:**

In all patients, fluorescence signal was detected in vivo during endoscopic SNIP surgery. Using ex vivo fluorescence grid analysis, we were able to correlate bevacizumab-800CW fluorescence of individual tissue fragments with final histopathology. Fluorescence grid analysis showed substantial variability in mean fluorescence intensity (*FI*_mean_), with SNIP tissue showing a median *FI*_mean_ of 77.54 (IQR 50.47–112.30) compared to 35.99 (IQR 21.48–57.81) in uninvolved tissue (*p* < 0.0001), although the diagnostic ability was limited with an area under the curve of 0.78.

**Conclusions:**

A fluorescence grid analysis could serve as a valid method to evaluate fluorescence molecular imaging in piecemeal surgeries. As such, although substantial differences were observed in fluorescence intensities, VEGF-A may not be the ideal target for SNIP surgery.

**Trial registration:**

NCT03925285.

**Supplementary Information:**

The online version contains supplementary material available at 10.1007/s00259-021-05567-x.

## Introduction

Sinonasal inverted papilloma (SNIP) is a benign tumor; yet, it is characterized by an aggressive growth pattern with destruction of adjacent bone, co-existing chronic inflammation, and risk of malignant transformation to squamous cell carcinoma [1, 2]. Even though the clinical features of SNIP are well described, the exact etiology of SNIP remains unclear [3]. The main treatment strategy of SNIP consists of endoscopic surgical resection to remove all SNIP tissue, and in more progressed stages even resecting the underlying bone at the insertion point to remove microscopic mucosal residues [1, 4]. Despite performing a thorough resection, local recurrence rates of up to 19% have been reported [5, 6], of which incomplete resection is considered to be the main causal factor [7]. This illustrates the unmet clinical need for tools that can improve visualization of all SNIP tissue in this anatomically complex area and aid in achieving a complete resection.

Fluorescence molecular imaging (FMI) is an emerging surgical guidance technique that has gained increasing interest in head and neck surgery [8]. FMI provides high-resolution visualization of disease-specific biomarkers and has been shown to adequately discriminate target tissue from surrounding tissues for various indications [9–13]. Therefore, it is of interest to evaluate the potential of FMI to highlight in vivo SNIP tissue intraoperatively and provide real-time feedback on excised tissue during SNIP surgery. The main biomarkers that have been associated with SNIP pathogenesis and may serve as a target for FMI include cyclooxygenase [14], epidermal growth factor [15, 16], and vascular endothelial growth factor (VEGF) [17–19]. It was shown that VEGF, a key mediator in angiogenesis related to inflammation and uncontrolled cell growth, correlated the Krouse classification system for assessment of resectability [19]. Consequently, targeted imaging of these biomarkers of SNIP might assist the surgeon in more complete resection. FMI using the near-infrared fluorescent tracer bevacizumab-800CW targeting the soluble ligand VEGF-A has been performed successfully for perioperative fluorescence detection and delineation in various solid tumors, including both malignant and benign tumors [20–23].

This study aimed to determine if the intravenously administered NIR fluorescent tracer bevacizumab-800CW accumulates in SNIP and can be used to discriminate between SNIP and uninvolved tissue, which may aid in radical resection of SNIP. Therefore, we studied the feasibility for clinical use of bevacizumab-800CW fluorescence both in vivo during endoscopic SNIP surgery and ex vivo by imaging the freshly excised tissue fragments after piecemeal surgery.

## Materials and methods

### Study design and patients

This proof-of-concept study was performed by the Departments of Otolaryngology and Oral & Maxillofacial Surgery of the University Medical Center Groningen (UMCG). Patients > 18 years old scheduled for surgical removal of histologically confirmed SNIP were enrolled in the study. The study was performed in concordance with the Declaration of Helsinki (adapted version Fortaleza, Brazil, 2013) and the Dutch Act on Medical Research involving Human Subjects. Approval was obtained at the Institutional Review Board of the UMCG (NL66494.042.18). Informed consent was obtained from all subjects prior to any study procedure. The trial was registered at www.clinicaltrials.gov (NCT03925285).

### The fluorescent tracer bevacizumab-800CW

Clinical-grade bevacizumab-800CW was manufactured in the good manufacturing practice unit of the UMCG, as previously described [24, 25]. Briefly, bevacizumab (Roche, AG) and IRDye-800CW-NHS (LI-COR Biosciences, NE, USA) were conjugated to a dye to antibody ratio of 2:1. Subsequently, bevacizumab-800CW was formulated in a sodium chloride solution at a concentration of 1 mg/mL. Based on prior clinical trial findings with the tracer [20, 22, 26], a dose of 10 mg was chosen for the current study, based on an optimal target-to-background ratio (TBR) obtained in vivo and limited variance in tumor *FI*_mean_.

**Fig. 1:**
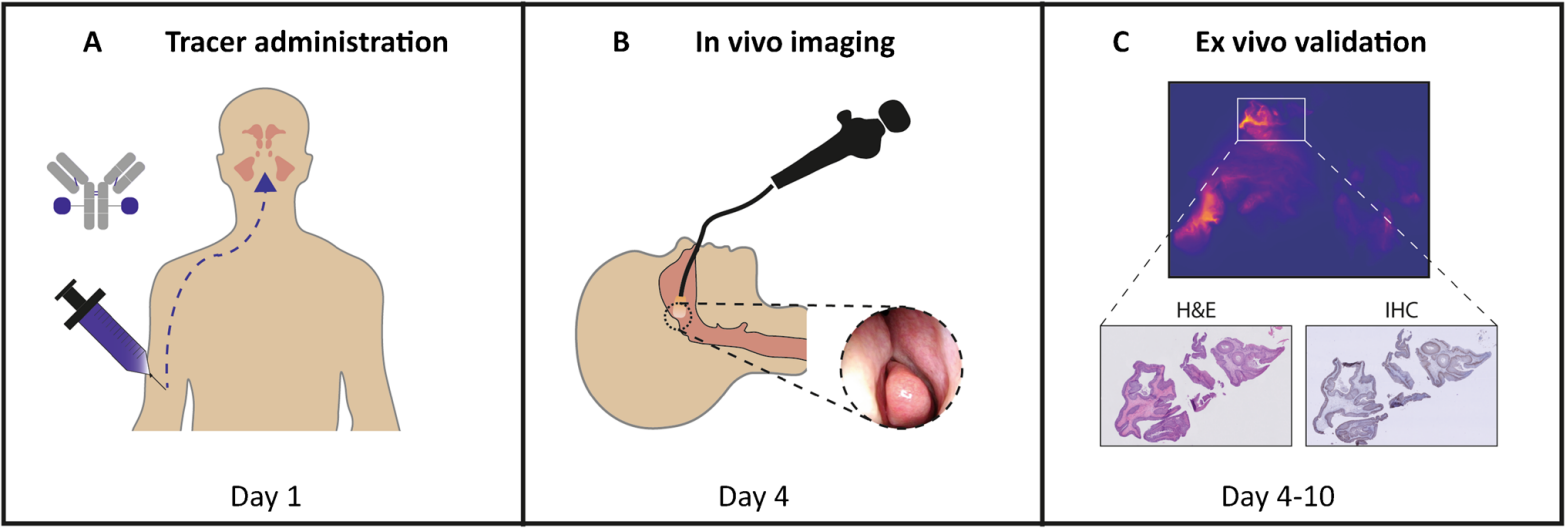
Summary of study workflow. **A** Bevacizumab-800CW is administered intravenously through a bolus injection. **B** Three days after administration, in vivo fluorescence molecular imaging is performed during surgery to study the clinical use. **C** During tissue processing, ex vivo fluorescence molecular imaging of all FFPE blocks is performed. Ultimately, fluorescence signal is correlated with final H&E histopathology (i.e., presence of SNIP) and VEGF-A immunohistochemistry. Abbreviations: FFPE, formalin-fixed, paraffin-embedded; H&E, hematoxylin and eosin; SNIP, sinonasal inverted papilloma; VEGF-A, vascular endothelial growth factor A

Three days prior to surgery, patients were administered a bolus injection of 10 mg bevacizumab-800CW intravenously. Patients were monitored after tracer administration for 1 h, and adverse events were reported according to the Common Terminology Criteria for Adverse Events (CTCAE) version 4.0. The study workflow is summarized in Fig. 1.

### Intraoperative imaging

All subjects underwent endoscopic surgery according to standard of care. Before resection, FMI of all locations (e.g., maxillary sinus, nasal cavity) comprising SNIP tissue was performed, using the contralateral (i.e., unaffected) side as a negative control. After resection, nasal pledgets soaked in cocaine/adrenaline were introduced to stop bleeding and temporarily removed to obtain fluorescence images of the wound beds. Immediately after resection, all tissue fragments including both SNIP and uninvolved tissue (i.e., normal mucosa, reactive mucosa, and connective tissue) were imaged during surgery at the back-table using a closed-field fluorescence imaging system (Pearl Trilogy, LI-COR Biosciences, NE, USA).

### Ex vivo* validation*

After surgery and closed-field imaging in the Pearl imaging system, all tissue fragments were submitted to the Department of Pathology, formalin-fixed and paraffin-embedded (FFPE) in tissue blocks. FFPE blocks were imaged with the Odyssey CLX® flatbed scanner (LI-COR Biosciences Inc., NE, USA) to further perform in-depth analysis of the localization of the fluorescence signals. Subsequently, 3-μm tissue sections were cut for hematoxylin and eosin (H&E) staining and were digitalized for further analysis.

**Fig. 2:**
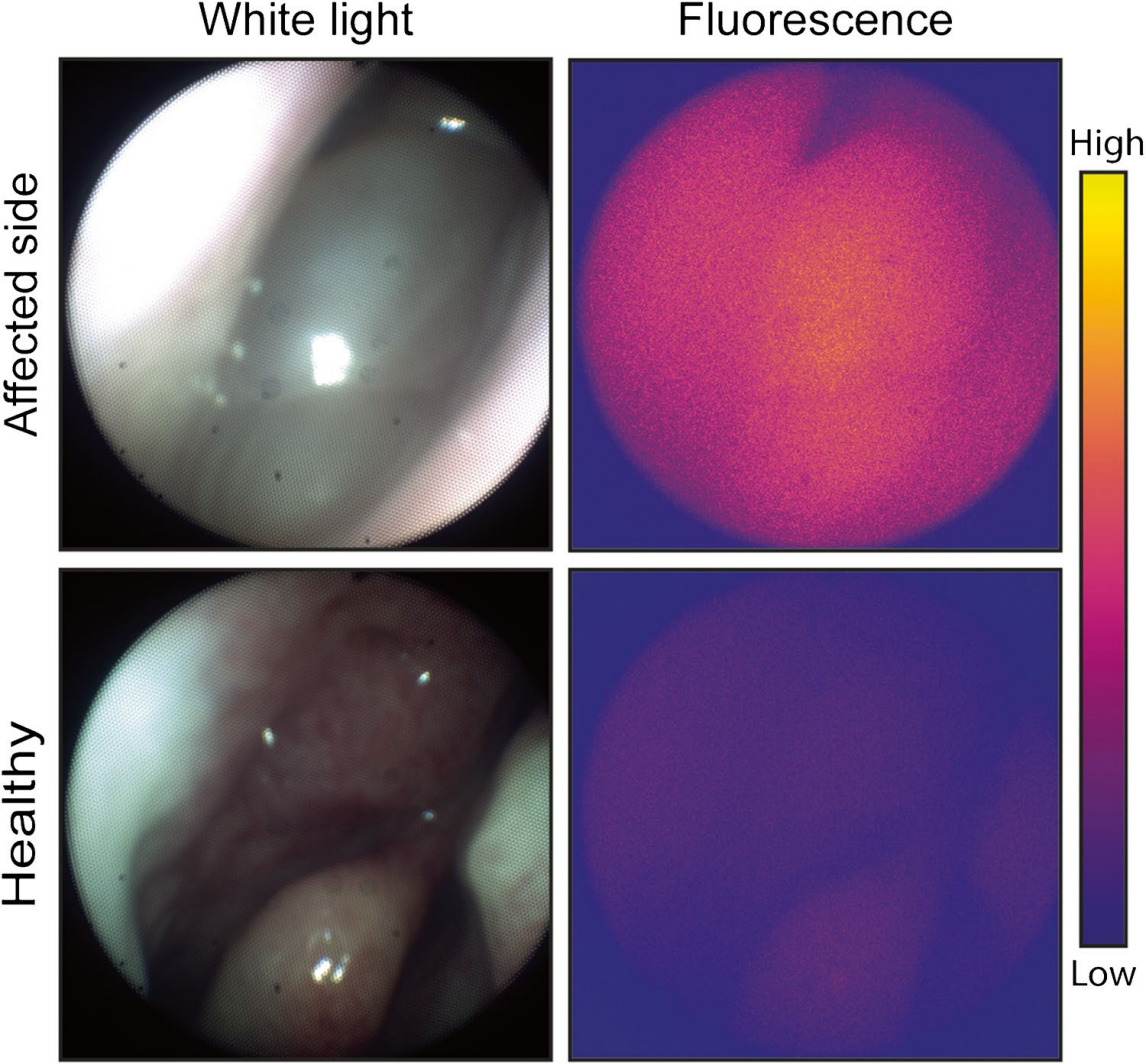
Ex vivo fluorescence molecular imaging analyses. **A** Using the standard fluorescence analysis, H&E segmentation of SNIP and uninvolved tissue are superimposed on the fluorescence image of the corresponding FFPE block. This method renders one value of both SNIP (blue ROI) and uninvolved tissue (orange ROI) per FFPE block, in which variability in fluorescence intensity is undesirably averaged. **B** In the fluorescence grid analysis, a 25 × 25 pixel grid is rendered on the fluorescence image. Using H&E segmentation as a reference, each square is scored as SNIP or uninvolved tissue, excluding squares that do not completely comprise tissue. Mean fluorescence intensity is calculated per square, resulting in multiple data points that demonstrate variability in signal. Abbreviations: FFPE, formalin-fixed, paraffin-embedded; SNIP, sinonasal inverted papilloma; TBR, target-to-background ratio

Our standard analysis of FMI consisted of drawing regions of interest (ROI) containing SNIP on the 3-μm H&E sections by a head and neck pathologist blinded for FMI results. ROIs were superimposed on the fluorescence flatbed scan of the corresponding FFPE block, and the *FI*_mean_ of SNIP and uninvolved tissue were determined (Fig. 2A). A TBR was calculated by dividing the target ROI (*FI*_mean_ SNIP) with the background ROI (*FI*_mean_ uninvolved tissue) per tissue slice. Median TBR values were presented per patient. Additional VEGF-A immunohistochemistry was performed per patient on two tissue sections that each contained both SNIP and uninvolved tissue. Fluorescence flatbed scans were used to correlate bevacizumab-800CW fluorescence and immunohistochemical VEGF-A expression qualitatively.

### Fluorescence grid analysis

Due to the nature of the surgical SNIP procedure, i.e., piecemeal resection, a requirement for the clinical use of FMI is identifying the nature of the small tissue fragments. Consequently, some of the tissue fragments contain normal tissue as well as SNIP or separately and not en bloc as seen in complete surgical resections. To better study the fluorescence signal within all tissue fragments, a grid of 25 × 25 pixels was used as an overlay for fluorescence images that have been obtained with the Odyssey CLX® flatbed scanner [30]. Each square was scored as either SNIP or uninvolved tissue based on the assessment of the pathologist (BvdV). Only the squares that completely comprised SNIP or uninvolved tissue were included in the analysis. The mean fluorescence intensity (*FI*_mean_) was automatically calculated with MATLAB (versionR2020a, The MathWorks, Natick, USA) for each square (Fig. 2B).

### Statistical analysis

Descriptive statistics were performed on patient demographics. Given the small sample size, individual data were presented in tabular formats. Due to the limited sample size, all data was considered non-normally distributed. Imaging data were presented as median with range or interquartile range (IQR). *FI*_mean_ was defined as total counts per ROI pixel area (signal/pixel), and calculated in ImageJ (Fiji, version 2.0.0) for standard fluorescence analysis. Fluorescence grid data was generated using MATLAB (versionR2020a, The MathWorks, Natick, USA), and the *FI*_mean_ per square was calculated automatically. For comparison of fluorescence imaging data, the Mann–Whitney *U* test was used. Cutoff values of fluorescence grid analysis were determined based on Youden’s *J* statistics. Sensitivity, specificity, and accuracy were calculated using standard formulas. A two-sided *p*-value < 0.05 was considered statistically significant. Statistical analyses and graph designs were performed using GraphPad Prism (version 9.0, GraphPad Software Inc., San Diego, CA, USA).

## Results

Between January 2019 and November 2020, six subjects were included in the study. One subject did not show SNIP on final histopathology and was used as a reference. No malignant transformation of SNIP was observed. Patient demographics and clinical features are summarized in Table 1. All patients received a single bolus injection of 10 mg bevacizumab-800CW without any side effects.

**Table 1:** Patient and clinical characteristics of study population

	1	2	3	4	5	6
Gender	Male	Female	Male	Male	Male	Male
Age	68	49	54	72	49	57
Insertion site	Nasal cavity	Unknown*	Ethmoid	Maxillary	Ethmoid	Nasal cavity
Number of prior surgeries	1	1	1	1	2	2
Surgical approach	Endoscopic	Endoscopic	Endoscopic	Endoscopic	Endoscopic	Endoscopic
Final pathology	SNIP	SNIP No	SNIP No	SNIP	Chronic inflammation	SNIP
Malignant transformation	No	No	No	No	No	No

^*^The insertion site could not be determined during surgery. *Abbreviations*: *SNIP*, sinonasal inverted papilloma

### In vivo* fluorescence-guided endoscopic surgery*

**Fig. 3:**
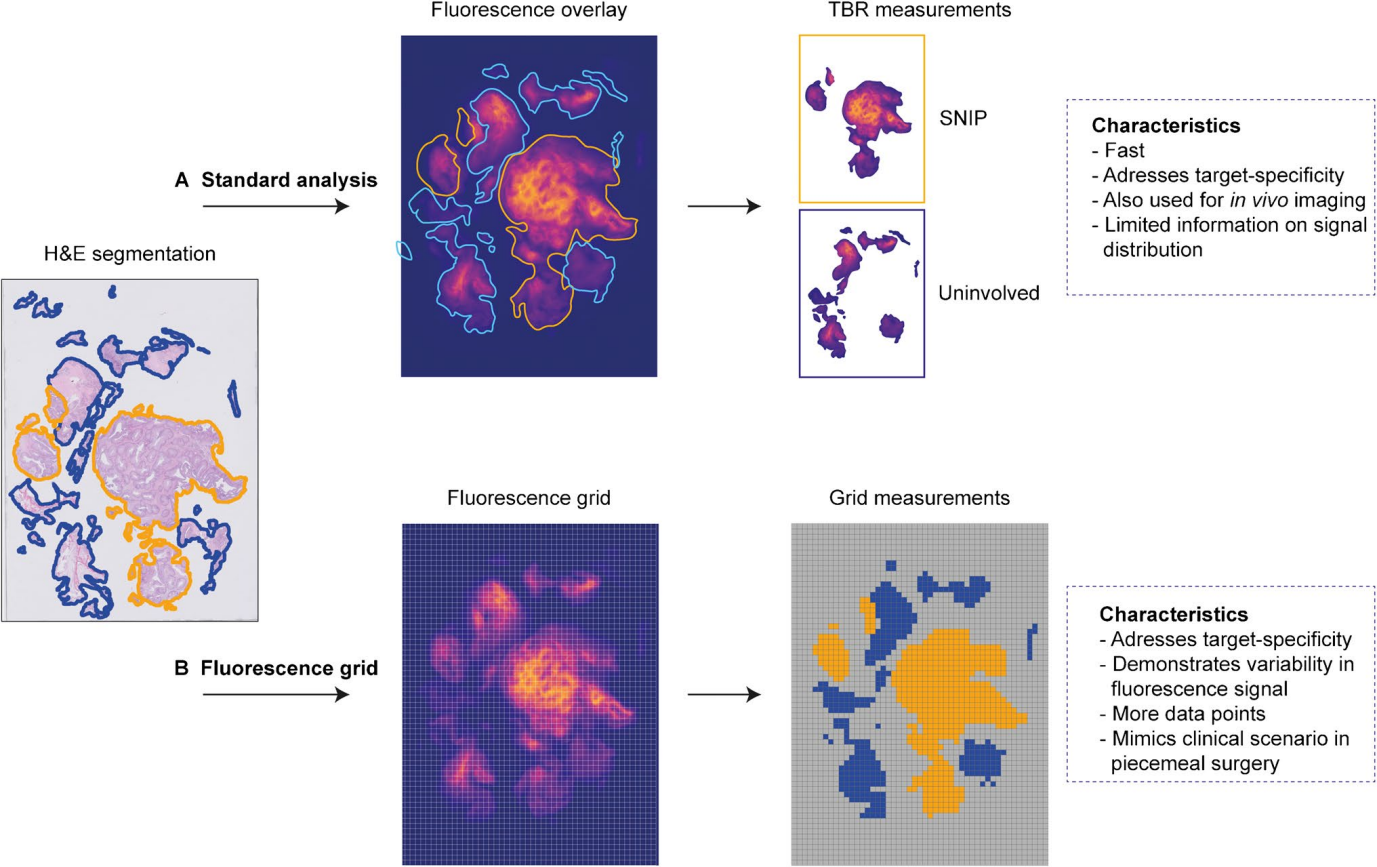
In vivo visualization of fluorescence during endoscopic sinus surgery. Representative images obtained with the nasoendoscopic fluorescence imaging system. The affected side shows higher fluorescence signal compared to the contralateral healthy side. Both inverted papilloma and chronic inflammation were observed at final histopathology, so it is unclear whether fluorescence signal at the affected side is specific for inverted papilloma

All five subjects underwent endoscopic endonasal surgery without conversion to an open procedure. In all cases, in vivo imaging was performed, and if applicable, multiple locations containing SNIP were inspected before resection. Endoscopic detection of fluorescence signal was possible in all patients; however, TBRs could not be calculated since SNIP could not be reliably differentiated from uninvolved tissue, which is a necessity for calculating tumor to normal ratios in vivo. Qualitatively, the affected side showed higher fluorescence than the healthy side for all five patients (Fig. 3). Imaging of the wound bed did not result in useable images for analysis due to the presence of blood.

### Ex vivo* fluorescence molecular imaging of bevacizumab-800CW*

**Fig. 4:**
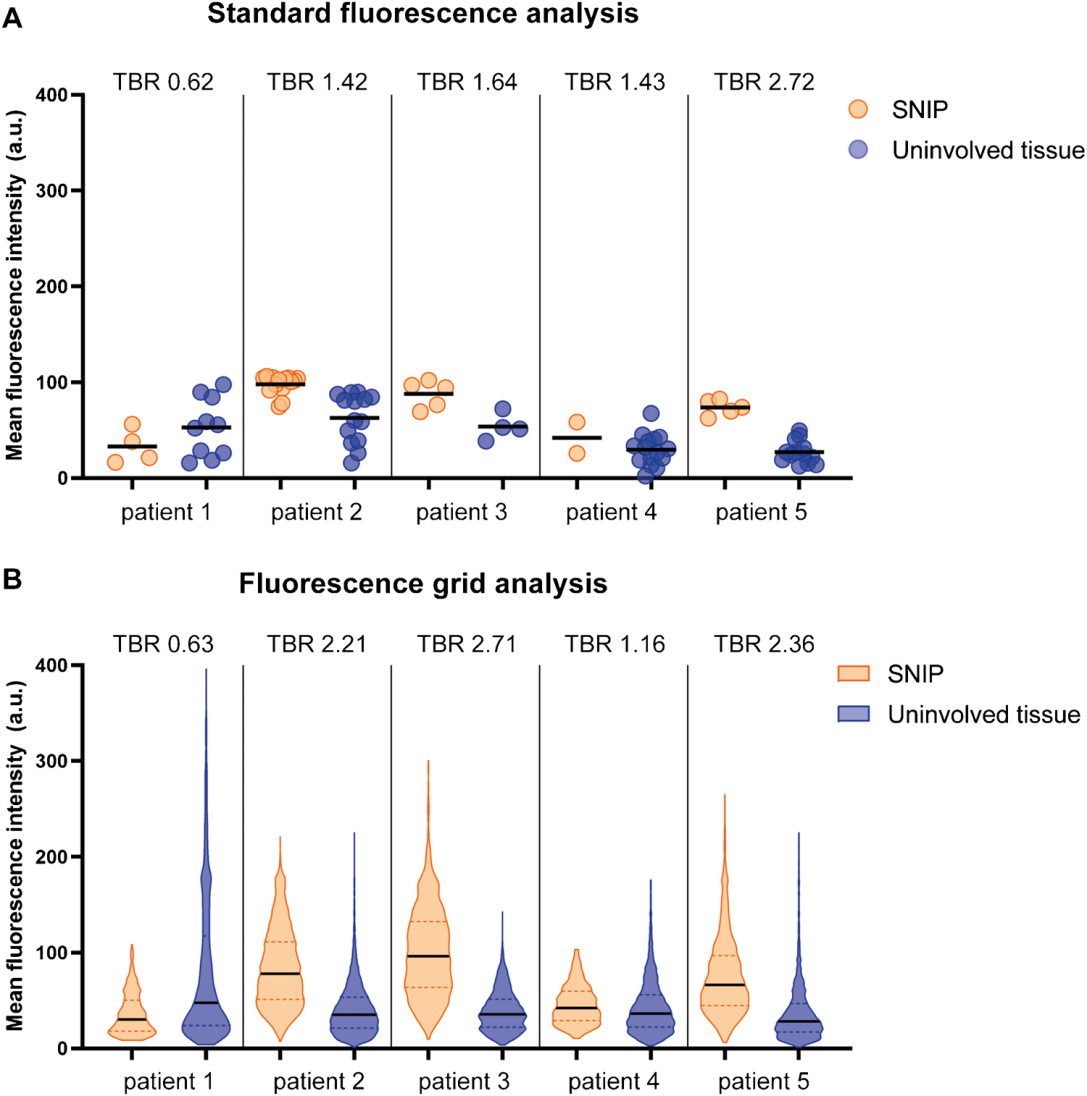
Fluorescence molecular imaging of formalin-fixed, paraffin-embedded tissue. **A** Scatter plot of the standard fluorescence analysis. Each circle represents the *FI*_mean_ of a single FFPE block (*n* = 61), with some FFPE blocks comprising both SNIP and uninvolved tissue. For each patient, the TBR is shown above the corresponding dots. Patient 1 did not show VEGF-A expression. **B** Violin plot of the *FI*_mean_ observed with fluorescence grid analysis. Because of the large amount of data points, data is visualized using violin plots instead of scatter plots. The *FI*_mean_ of all squares (*n* = 30,425) comprising either SNIP or uninvolved mucosa are shown. Albeit the TBRs of the fluorescence grid analysis are different from standard fluorescence analysis, the main difference between the two analysis methods is that the fluorescence grid analysis better shows the variability in fluorescence intensity. As such, whereas both methods show a notable difference in fluorescence intensity between SNIP and uninvolved mucosa, the fluorescence grid analysis better evaluates the imaging approach in the light of piecemeal surgery, which requires assessment of individual tissue fragments. Abbreviations: *FI*_mean_, mean fluorescence intensity; FFPE, formalin-fixed, paraffin-embedded; TBR, target-to-background ratio; VEGF-A, vascular endothelial growth factor A; SNIP, sinonasal inverted papilloma

To determine the potential of bevacizumab-800CW for the discrimination between SNIP and uninvolved tissue, we calculated the *FI*_mean_ of the FFPE tissue blocks (*n* = 61). As most tissue sections contained both SNIP and uninvolved tissue, a total of 30 FFPE blocks containing SNIP and 52 containing uninvolved tissue were identified. Median *FI*_mean_ in SNIP was 86.88 (IQR 67.57–101.20) compared to 38.30 (IQR 22.28–57.99) in uninvolved tissue (*p* < 0.0001), although substantial variation was observed within and between patients. The median *FI*_mean_ and corresponding TBRs per patient are shown in Fig. 4A.

We studied the variability of fluorescence signal between different tissue fragments in more detail using the 25 × 25 pixel grid analysis. A total of 202,752 grid squares was rendered, of which 30,425 completely comprised tissue, with 13,454 classified as SNIP and 28,025 as uninvolved tissue based on H&E histopathology. As such, 2973–22,062 measurements per patient were obtained to study the fluorescence signal (Fig. 2B). Again, higher median *FI*_mean_ was observed in SNIP; 77.54 (IQR 50.47–112.30) compared to uninvolved tissue 35.99 (IQR 21.48–57.81) (*p* < 0.0001). Even though similar TBRs were obtained, the fluorescence grid analysis showed greater variability in fluorescence intensity (Fig. 4B). The ROC curve for all patients combined showed an area under the curve of 0.78 (Supplemental Fig. 1). The optimal cutoff value of 52.80 *FI*_mean_ rendered 72.71% sensitivity and 70.72% specificity, with a positive predictive value and negative predictive value of 54.39% and 84.37%, respectively.

### Distribution of bevacizumab-800CW in SNIP at a microscopic level

**Fig. 5:**
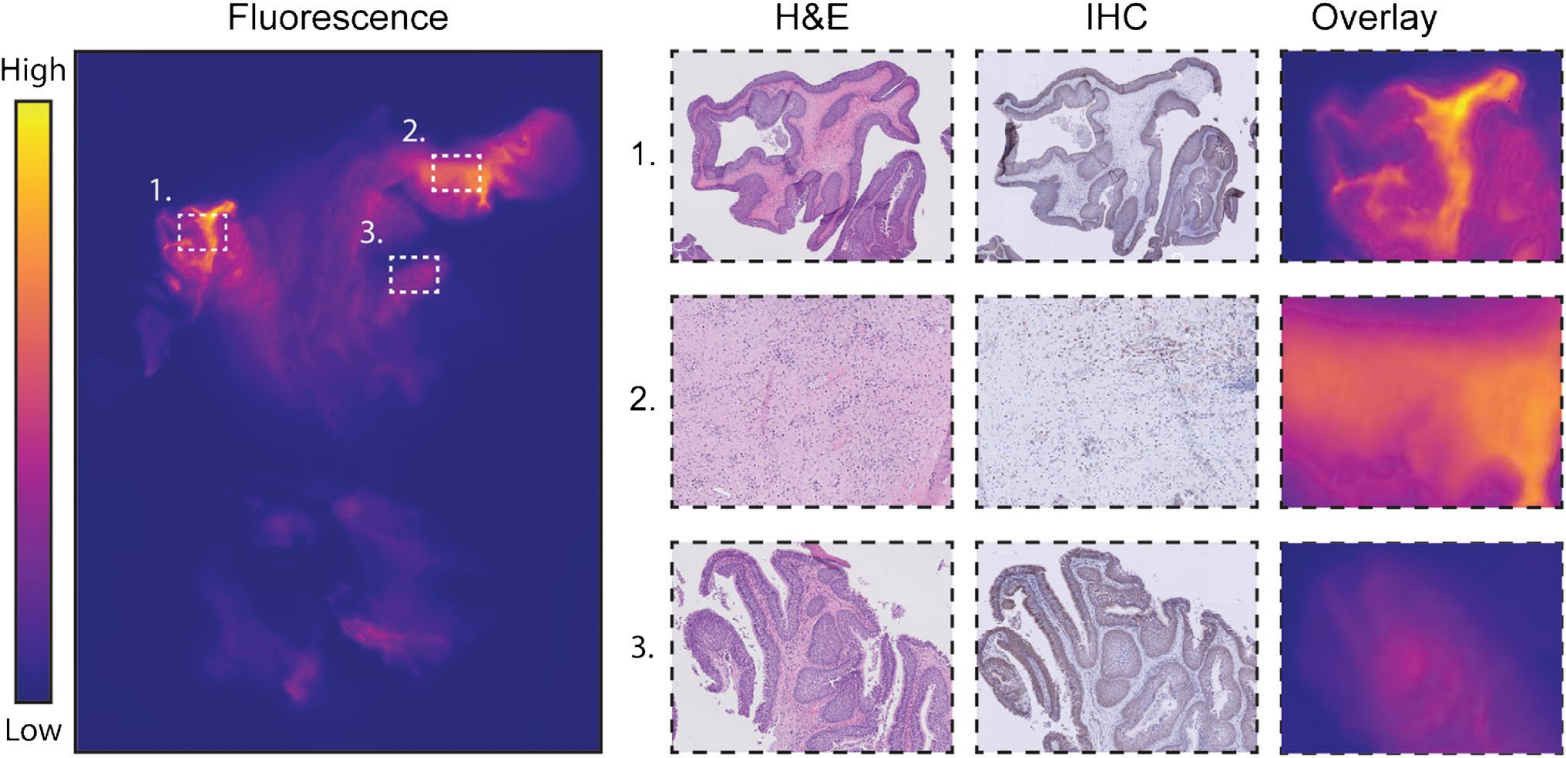
Ex vivo validation. To study the microscopic tracer distribution of bevacizumab-800CW, fluorescence images were correlated with corresponding VEGF-A IHC and H&E tissue sections. We observed an increased fluorescence signal in regions showing VEGF-A expression. These regions were not only limited to SNIP (1) but also included reactive stroma and submucosa with an abundance of plasma cells that expressed VEGF-A (2). Variability in fluorescence signal was observed between different regions of SNIP (1, 3). In addition, regions including edema and increased vasculature also showed increased fluorescence signal despite absence of VEGF expression (not shown in figure). Abbreviations: VEGF-A, vascular endothelial growth factor A; IHC, immunohistochemistry; H&E, hematoxylin and eosin

VEGF immunohistochemistry of tissue sections was performed and compared to fluorescence flatbed scans of the corresponding FFPE-block to study co-localization of bevacizumab-800CW with VEGF-A expression. Four of five patients showed VEGF-A expression. Increased fluorescence signal was observed in, but not limited to, regions showing VEGF-A expression. The regions that showed increased VEGF-A expression were not only limited to SNIP but also included reactive stroma and submucosa with an abundance of plasma cells in VEGF-A positive stroma. In addition, regions showing edema and increase in microvasculature showed increased fluorescence signal despite absent or low VEGF-A expression. A representative image of VEGF-A immunohistochemistry and co-localization with fluorescence flatbed scanning is shown in Fig. 5.

## Discussion

To our knowledge, this is the first clinical study that investigates the potential of FMI in SNIP using the VEGF-targeted antibody bevacizumab-800CW. Here, we show an increased fluorescence signal in SNIP compared to uninvolved tissue, when using both the standard fluorescence analysis and the fluorescence grid analysis. In addition, all patients displayed visually more in vivo fluorescence signal of the affected side compared to the contralateral healthy side. However, it was not possible to attribute the observed higher fluorescence signal during endoscopic imaging to SNIP because SNIP could not be differentiated from adjacent inflammatory mucosa based on clinical assessment.

Throughout the current study, we have used several outcome parameters to study FMI using bevacizumab-800CW in SNIP. First, we obtained the in vivo FMI contrast to study the potential of intraoperative use. Although the in vivo target-to-background contrast in FMI is most likely the ultimate clinical application, evaluation of the fluorescent tracer for a particular indication is preferably performed in an ex vivo environment. This allows for optimal control of imaging parameters (i.e., camera-tissue distance, angle of illumination, and ambient light) [27]. Here, the observed differences in fluorescence signal can be attributed more realistically to tissue tracer distribution rather than differences in imaging parameters. To establish a tool that can be used for intraoperative clinical decision-making, we aimed to study the use of back-table FMI to identify SNIP in the freshly excised tissue fragments. As the tissue fragments’ position could only be fixed at the last step of the tissue processing procedure, i.e., during embedding in a paraffin block, it was impossible to keep track of the individual fragment’s position during the earlier steps of the formalin fixation and paraffin embedding. Consequently, we were not able to correlate back-table FMI with final histopathology. Therefore, we validated the FMI by correlating ex vivo imaging of FFPE blocks with H&E histopathology and target expression (i.e., VEGF-A immunohistochemistry). Earlier, it has been shown that the processing of tissue does not alter relative differences in fluorescence between target and uninvolved tissues [28], suggesting that the fluorescence grid analysis can help in evaluating FMI for clinical use, thereby providing substantiated go/no-go decisions in phase I/II studies. We ascertained that the standard method of evaluating FMI for wide local excisions is not preferable for piecemeal surgery since it masks the (substantial) variability present in the imaging data as only two ROIs (i.e., target and background) are obtained per FFPE block. Therefore, we have developed a fluorescence grid analysis that may serve as a structured method for evaluating novel compounds in early-phase FMI studies in piecemeal surgery without interfering with the current analytical frameworks used for FMI.

Although multiple clinical studies have shown successful use of FMI in the removal of solid tumors, piecemeal surgery may require a different approach. The microscopically heterogeneous expression of the target that is typically observed does not per se impede guiding the surgeon in resection of bulk tissue (i.e., wide local excision) due to the margin of primary interest. In contrast, in piecemeal surgery, removal of microscopic residues is more critical and macroscopic imaging of a heterogeneous pattern may be insufficient. Here, microscopic small tissue fragments must be independently assessed for the presence of the disease (i.e., SNIP in the current study). Therefore, we have developed a novel method for the evaluation of FMI in piecemeal surgeries using a fluorescence grid analysis. Although in this study the TBRs of the fluorescence grid analysis were not substantially different from those of the standard analysis, the former method provided better insight in the variability of fluorescence signal. When evaluating FMI in piecemeal surgery, this variability of data is very important as the ultimate goal is to identify all tissue fragments that comprise the target tissue. When a suitable biomarker is identified through fluorescence grid analysis, the clinical value (i.e., in vivo contrast) can be assessed in a subsequent study.

Other studies that address the clinical problem of incomplete resection of SNIP have also reported the use of alternative imaging techniques to discriminate between SNIP and uninvolved tissue [29, 30]. Yet, these imaging methods are mainly limited to morphological information and therefore lack specificity to distinguish SNIP from surrounding inflammatory tissue. Molecular imaging approaches may improve the specificity of contrast and thus harbor more clinical potential. Recently, the use of Raman spectroscopy showed 90% accuracy in distinguishing normal sinonasal mucosa, chronic rhinosinusitis, and SNIP tissue [31]. In contrast to Raman spectroscopy, a point measurement spectroscopy technique, FMI can visualize the complete mucosa of interest in vivo and can guide the surgeon during SNIP surgery rather than measure the surgeon’s observations.

In the current study, considerable variability in fluorescent intensity was observed in both SNIP and uninvolved mucosa. Fluorescence signal was not only limited to SNIP but also occurred in regions that showed abundancy of plasma cells expressing VEGF-A and tissue showing reactive changes and edema. This moderate co-localization of bevacizumab-800CW and VEGF-A expression is in line with previous studies [21, 23, 32]. We surmise that this is because bevacizumab-800CW targets the extracellular matrix-bound isoform of VEGF-A, whereas immunohistochemical staining mainly detects an intracellular VEGF-A isoform [33, 34]. Secondly, and often underestimated in targeted imaging, nonspecific mechanisms, such as vascularity, vascular permeability, interstitial pressure, and internalization of the tracer, contribute to accumulation of the fluorescent tracer in the target tissue [35–37]. For instance, the increase in VEGF-A may cause increased vascular permeability in the inverted papilloma and adjacent inflammatory tissue [38, 39]. Differentiating between nonspecific and specific accumulation of fluorescent tracers is one of the most important challenges in FMI today. Efforts that may solve this problem to better quantify receptor expression include paired-imaging agent strategies, which are now evaluated in preclinical studies [40, 41]. Although previous studies with bevacizumab-800CW showed tumor-specific fluorescence signal [10, 20, 22], we believe that for piecemeal SNIP surgery (in vivo) FMI using bevacizumab-800CW lacks sensitivity and specificity for SNIP surgery, which limits clinical applicability.

The performance of FMI in SNIP surgery can be improved in two ways. First, improved understanding of the poorly understood SNIP pathogenesis may help identify new biomarkers that are highly specific for SNIP tissue. Proteins involved in epithelial remodeling into SNIP may be of interest, of which the main etiologies include human papillomavirus [42, 43], chronic inflammation [44, 45], and angiogenic factors [19]. To further increase the possibility of detecting small SNIP residues, fluorescent tracers with an on/off mechanism could be exploited to achieve fluorescence signal in the target tissue only and increase contrast ^45^. Technical challenges for in vivo endoscopic FMI remain due to a narrow field of view in the sinonasal anatomical area and the substantial bleeding that occurs during SNIP surgery, which obscures the optical signal and impedes in vivo contrast.

In conclusion, although SNIP tissue showed increased fluorescence signal compared to uninvolved mucosa, the clinical applicability of VEGF-targeted FMI is limited by low sensitivity and specificity to differentiate small tissue fragments. To overcome the problem of ex vivo tissue fragment analysis that occurs after piecemeal surgery, we have developed fluorescence grid analysis, a novel framework for the assessment of FMI. This method may serve as a guideline for future studies that evaluate FMI in piecemeal surgery using other disease targets. When more specific biomarkers for SNIP are discovered, a fluorescence grid analysis could more comprehensively inform about the clinical applicability of the fluorescent tracer studied in phase I studies, allowing for go/no-go decision in an early stage.

## Electronic supplementary material

Figure S1  **ROC curve of fluorescence grid analysis**. ROC curve of all patients combined based on mean fluorescence intensity (FImean) as determined with fluorescence grid analysis shows an area under the curve of 0.78. (PNG 89.4 kb)

High Resolution Image (TIF 265 kb)

## Data Availability

All data are available from the corresponding author (MJHW) on reasonable request.
