# Supplementary figures and images for: Fluorescence grid analysis for the evaluation of piecemeal surgery in sinonasal inverted papilloma: a proof-of-concept study
Source: Eur J Nucl Med Mol Imaging. 2021 Nov 5;49(5):1640–9. doi: 10.1007/s00259-021-05567-x (PMC8940828; doi:10.1007/s00259-021-05567-x)

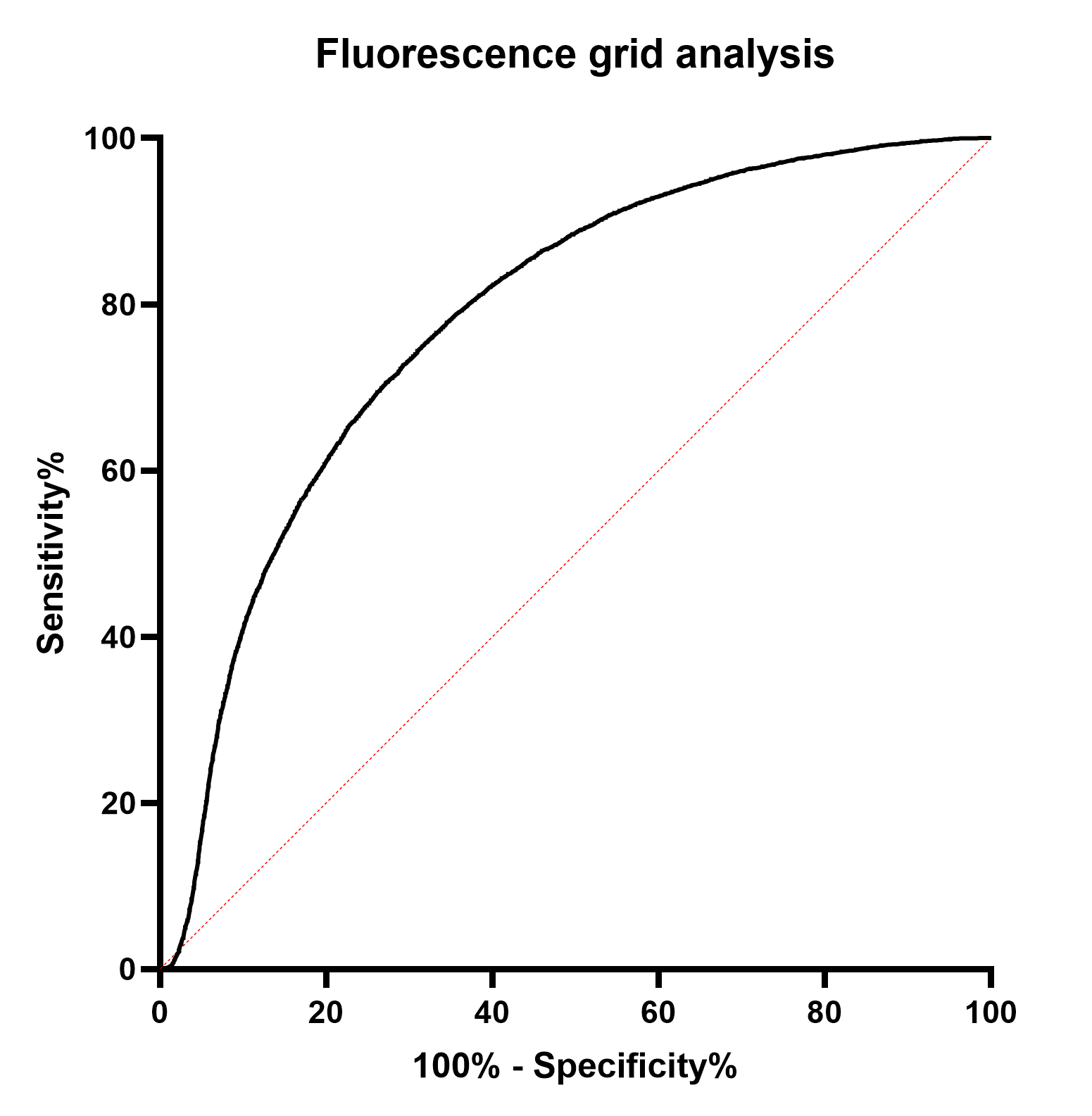

Supplement: Supplementary file 1 — ROC curve of fluorescence grid analysis. ROC curve of all patients combined based on mean fluorescence intensity (FImean) as determined with fluorescence grid analysis shows an area under the curve of 0.78. (PNG 89.4 kb) [file 259_2021_5567_Fig6_ESM.png]

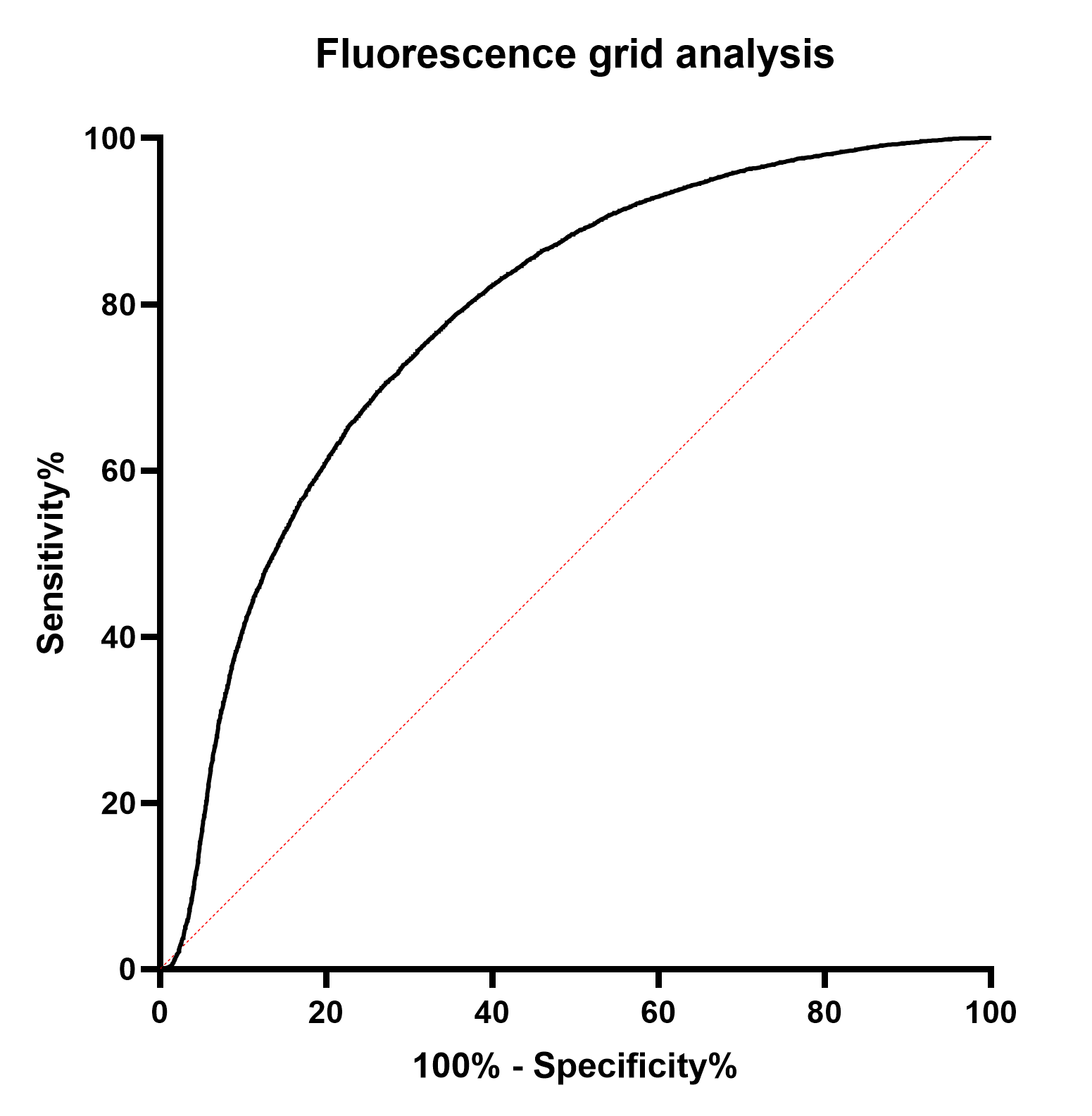

Supplement: Supplementary file 2 — High Resolution Image (TIF 265 kb) [file 259_2021_5567_MOESM1_ESM.tif]
